# Differential Genetic and Epigenetic Regulation of catechol-*O*-methyltransferase is Associated with Impaired Fear Inhibition in Posttraumatic Stress Disorder

**DOI:** 10.3389/fnbeh.2013.00030

**Published:** 2013-04-10

**Authors:** Seth Davin Norrholm, Tanja Jovanovic, Alicia K. Smith, Elisabeth Binder, Torsten Klengel, Karen Conneely, Kristina B. Mercer, Jennifer S. Davis, Kimberly Kerley, Jennifer Winkler, Charles F. Gillespie, Bekh Bradley, Kerry J. Ressler

**Affiliations:** ^1^Mental Health Service Line, Atlanta VA Medical CenterDecatur, GA, USA; ^2^Department of Psychiatry and Behavioral Sciences, Emory University School of MedicineAtlanta, GA, USA; ^3^Max Planck Institute of PsychiatryMunich, Germany; ^4^Department of Human Genetics, Emory University School of MedicineAtlanta, GA, USA; ^5^Howard Hughes Medical InstituteChevy Chase, MD, USA

**Keywords:** catechol-*O*-methyltransferase, fear-potentiated startle, posttraumatic stress disorder, epigenetic, methylation, trauma

## Abstract

The catechol-*O*-methyltransferase (COMT) enzyme is critical for the catabolic regulation of synaptic dopamine, resulting in altered cortical functioning. The *COMT* Val^158^Met polymorphism has been implicated in human mental illness, with Met/Met homozygotes associated with increased susceptibility to posttraumatic stress disorder (PTSD). Our primary objective was to examine the intermediate phenotype of fear inhibition in PTSD stratified by *COMT* genotype (Met/Met, Val/Met, and Val/Val) and differential gene regulation via methylation status at CpG sites in the *COMT* promoter region. More specifically, we examined the potential interaction of *COMT* genotype and PTSD diagnosis on fear-potentiated startle during fear conditioning and extinction and *COMT* DNA methylation levels (as determined using genomic DNA isolated from whole blood). Participants were recruited from medical and gynecological clinics of an urban hospital in Atlanta, GA, USA. We found that individuals with the Met/Met genotype demonstrated higher fear-potentiated startle to the CS− (safety signal) and during extinction of the CS+ (danger signal) compared to Val/Met and Val/Val genotypes. The PTSD+ Met/Met genotype group had the greatest impairment in fear inhibition to the CS− (*p* = 0.006), compared to Val carriers. In addition, the Met/Met genotype was associated with DNA methylation at four CpG sites, two of which were associated with impaired fear inhibition to the safety signal. These results suggest that multiple differential mechanisms for regulating COMT function – at the level of protein structure via the Val^158^Met genotype and at the level of gene regulation via differential methylation – are associated with impaired fear inhibition in PTSD.

## Introduction

Posttraumatic stress disorder (PTSD), a debilitating psychiatric illness precipitated by exposure to a traumatic event, occurs in approximately one-fourth of traumatized individuals (Institute of Medicine, 2008). As such, there is a compelling rationale for examining candidate risk and resilience factors for developing this disorder after experiencing trauma. Recent reviews in the literature have identified a number of candidate gene variants as potential contributors to PTSD risk and resilience (Amstadter et al., 2009; Jovanovic and Ressler, 2010; Skelton et al., 2012).

An intriguing, well-described candidate is the gene that encodes catechol-*O*-methyltransferase (*COMT*), an enzyme that catabolizes catecholamines such as norepinephrine, epinephrine, and dopamine. *COMT* is primarily expressed in the prefrontal cortex (Gogos et al., 1998; Tunbridge et al., 2004a) and hippocampus (Matsumoto et al., 2003), regions that are critically associated with inhibition of fear responses. The COMT enzyme represents the principal synaptic dopamine clearing mechanism in these brain regions that are largely devoid of dopamine transporter expression (Sesack et al., 1998; Lewis et al., 2001). The *COMT* gene is located on chromosome 22q11 and possesses several common single nucleotide polymorphisms (SNPs), including a G/A substitution (rs4680) at codon 158 in membrane bound COMT (MB-COMT) and codon 108 in soluble COMT (S-COMT). This SNP results in a valine (Val) to methionine (Met) substitution that affects the thermostability and activity of COMT. Met/Met homozygotes exhibit 35–50% less activity compared to Val/Val homozygotes (Chen et al., 2004); a decrement believed to increase brain dopamine levels in Met158 allele carriers (Bilder et al., 2004; Chen et al., 2004; Tunbridge et al., 2004b), and presumably, downstream alterations in dopamine receptor (primarily D1 in cortical regions) availability (Slifstein et al., 2008).

From a psychiatric perspective, Met allele carriers, and especially Met/Met homozygote individuals, may be more susceptible to anxiety disorders (Enoch et al., 2003; Woo et al., 2004; Olsson et al., 2005). This susceptibility is believed to be associated with greater responsiveness and increased connectivity between limbic brain regions such as the amygdala, hippocampus, and prefrontal cortex (Heinz and Smolka, 2006), and this has been supported by empirical data. For example, fMRI studies have reported greater limbic and prefrontal activation in Met allele carriers when exposed to negative imagery (Smolka et al., 2005; Drabant et al., 2006). In addition, the acoustic startle response, a reflexive contraction of the facial musculature (e.g., *orbicularis oculi*) in response to a sudden acoustic stimulus, may be enhanced in Met/Met homozygotes as compared to Val carriers. This enhancement has been observed upon exposure to aversive imagery (Montag et al., 2008), but also across negative, positive, neutral, and baseline conditions (Armbruster et al., 2011). Finally, a recent study that examined fear conditioning and extinction of fear-potentiated startle in healthy volunteers found less fear extinction in Met/Met homozygous individuals relative to Val carriers (Lonsdorf et al., 2009).

The potential contribution of genetic variation in *COMT* to PTSD susceptibility is being increasingly investigated. Recent work has implicated the *COMT* Val^158^Met polymorphism in reduced resilience (Armbruster et al., 2012), and the development of PTSD following repeated traumatic exposure (Kolassa et al., 2010). A recent study by Valente et al. (2011) of urban violence in Brazil identified a significant relationship between the Met158 allele and the development of PTSD following a violent encounter.

Interestingly, the Val^158^Met polymorphism also influences DNA methylation patterns at the *COMT* locus. Methylation of CpG sites, or genomic regions in which a phosphodiester bond links cytosine and guanine base pairs, can subsequently influence gene expression patterns and protein function (Jones and Takai, 2001). Indeed, stress-related methylation of the Val^158^Met polymorphism has already been linked to brain activity (Ursini et al., 2011). The regulation of *COMT* expression by both genetic and epigenetic factors may represent a potential mechanism underlying reported interactions between this polymorphism and environmental factors and warrant further attention. The purpose of the present study was to examine fear conditioning and extinction in a traumatized urban population with the study sample stratified by *COMT* genotype (Met/Met homozygotes, Val/Met heterozygotes, and Val/Val homozygotes) using an established fear-potentiated startle paradigm (Norrholm et al., 2011). In addition, we sought to examine methylation status at CpG sites in the promoter region of the *COMT* gene.

Our previous studies with fear-potentiated startle in PTSD have indicated that the fear-related symptoms of PTSD are associated with impaired fear inhibition (see Jovanovic and Norrholm, 2011). Although COMT has been extensively investigated in the literature, there are no studies that have specifically examined the relationship between the *COMT* genotype and methylation using psychophysiological measures of fear inhibition in PTSD. Based on our work in PTSD populations and previous studies of *COMT* genetic variation in fear extinction (Lonsdorf et al., 2009), we hypothesized that *COMT* Met158 homozygotes would display increased fear-potentiated startle to a safety signal and during extinction, and that this deficit in fear inhibition would be associated with PTSD.

## Materials and Methods

### Participants

Participants were recruited as part of a larger study investigating the genetic and environmental factors that contribute to PTSD in a primarily African American, low socioeconomic, inner-city population in Atlanta, GA, USA as well as from the Emory University community. Exclusion criteria included active psychosis and major medical illnesses as assessed by history and physical examinations, as well as hearing impairment assessed with an audiometer (Grason-Stadler, GS1710). Prior to their participation, all participants provided written informed consents approved by the Emory University Institutional Review Board and the Grady Health Systems Research Oversight Committee. Participants were assessed for demographic data, trauma history (using the Traumatic Events Interview, TEI, see Table 2), and PTSD symptoms (using the modified PTSD Symptom Scale, PSS). These measures have all been validated previously in this population (Binder et al., 2008). PTSD diagnosis was based on DSM-IV criteria using the PSS.

### Fear-potentiated startle assessment

Startle response data were acquired at a 1000 Hz sampling frequency using the electromyography (EMG) module of the BIOPAC MP150 for Windows (Biopac Systems, Inc., Aero Camino, CA, USA). The eyeblink component of the acoustic startle response was measured by EMG recordings of the right *orbicularis oculi* muscle using two 5-mm Ag/AgCl electrodes filled with electrolyte gel. One electrode was positioned 1 cm below the pupil of the right eye and the other was placed 1 cm below the lateral canthus. Impedance levels were less than 6 kΩ for each participant. The acquired data were filtered, rectified, and smoothed using the MindWare software suite (MindWare Technologies, Ltd., Gahanna, OH, USA) and exported for statistical analyses. The EMG signal was filtered with low- and high-frequency cutoffs at 28 and 500 Hz, respectively. The maximum amplitude of the eyeblink muscle contraction 20–200 ms after presentation of the startle probe was used as a measure of the acoustic startle response. The startle probe was a 106-dB (A) SPL, 40-ms burst of broadband noise with near instantaneous rise time, delivered binaurally through headphones.

**Figure 1:**
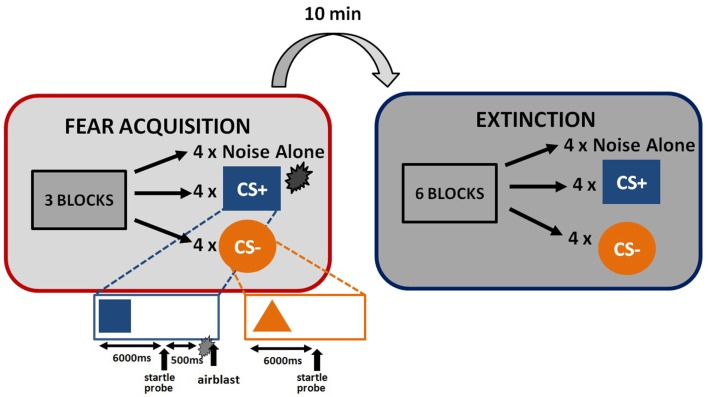
**Schematic illustration of fear-potentiated startle paradigm and the conditioned stimuli**.

The startle session began with a habituation phase to reduce startle reactivity and familiarize the subjects to the CSs. The fear conditioning phase immediately followed habituation and consisted of three blocks with four trials of each type (a reinforced conditioned stimulus, CS+; a non-reinforced conditioned stimulus, CS−; and the 40 ms, 108 dB noise probe alone (noise alone, NA), for a total of 36 trials. As expected, the CS+ acquires excitatory properties, signals the presence of an aversive outcome, and is termed a danger signal whereas the CS− acquires inhibitory properties, signals the lack of an aversive outcome, and is termed a safety signal (Norrholm et al., 2006). The use of this type of differential conditioning allows investigators to determine one’s ability to discriminate between danger and safety, most notably in patient populations with anxiety disorders (e.g., Norrholm et al., 2011). Figure 1 shows the schematic diagram of the stimuli in the experiment. Both CSs were colored shapes presented on a computer monitor for 6 s. The US was a 250 ms air blast with an intensity of 140 p.s.i. directed at the larynx. This US has been used in several of our previous studies and consistently produces robust fear-potentiated startle (Jovanovic et al., 2010b; Norrholm et al., 2011). For CS+ trials, a colored shape was displayed for a total of 6790 ms. A startle probe (40 ms duration) was administered 6000 ms after onset of the shape. The airblast US (250 ms duration) was then administered 500 ms after the startle probe. The shape display then co-terminated with the offset of the airblast. For CS− trials, a colored shape was displayed for a total of 6040 ms. A startle probe (40 ms duration) was administered 6000 ms after onset of the shape. The shape display then co-terminated with the offset of the startle probe. The inter-trial intervals were randomized to be 9–22 s in duration. The extinction session was administered 10 min after fear conditioning, and consisted of six blocks with four trials of each type (CS+, CS−, and NA), for a total of 72 trials. The stimuli were same as above, except that the CS+ was no longer reinforced with the airblast. The 10-min interval between the acquisition and extinction sessions was based on our previous work with healthy controls (Norrholm et al., 2008) and PTSD patient populations (Norrholm et al., 2011) and is consistent with similar work from other laboratories examining fear extinction and PTSD (Milad et al., 2008). Our data have shown that fear extinction performed 10 min after fear acquisition, as opposed to longer durations such as 72 h, does not affect extinction learning (Norrholm et al., 2008).

### US-expectancy

A response keypad (SuperLab, Cedrus Corp., San Pedro, CA, USA) was used during each acoustic startle session to record the participants’ expectancy of the US on each CS presentation (Jovanovic et al., 2006). Participants rated their expectancy of the airblast US by pressing one of three buttons: a button marked “+” if they expected the US on a given CS trial, a button marked “−” if they did not expect the airblast US, or a button marked “0” if they were uncertain. For data analysis purposes, responses of “+” were scored as 1, responses of “−” were scored as −1, and responses of “0” were scored as 0.

### Genotyping

DNA was extracted from saliva in Oragene collection vials (DNA Genotek Inc, ON, Canada) using the DNAdvance kit (Beckman Coulter Genomics, Danvers, MA, USA). The *COMT* Val^158^Met SNP, rs4680, was genotyped using the Sequenom iPlex chemistries and the MassARRAY system (Sequenom Inc., San Diego, CA, USA). The assay call rate was 97.6%. Within and across plate duplicates were used for quality control. All duplicates were concordant. Genotypes for control samples (identified as those without PTSD) were in Hardy–Weinberg Equilibrium (*p* > 0.05).

### Methylation

Genomic DNA was isolated from whole blood stored in EDTA tubes using the Gentra Puregene Kit (Qiagen, Hilden, Germany). DNA was quantified using PicoGreen (Invitrogen, Carlsbad, CA, USA), and the quality was checked on an agarose gel. CpG sites in *COMT* were selected from the HumanMethylation 450K BeadChip (Illumina, San Diego, CA, USA). Briefly, 1 μg of DNA converted with sodium bisulfite, amplified, fragmented, and hybridized on the HumanMethylation 450K BeadChip (Illumina, San Diego, CA, USA) according to the manufacturer’s instructions. Beta values were generated with Beadstudio. Samples with probe detection call rates <90% and those with an average intensity value of either <50% of the experiment-wide sample mean or <2000 arbitrary units (AU) were excluded from further analysis. One sample of male DNA was included on each BeadChip as a technical control throughout the experiment and assessed for reproducibility using the Pearson correlation coefficient. For each individual sample and CpG site, the signals from methylated (M) and unmethylated (U) bead types were used to calculate a beta value as β = M/(U + M). The methylation differences were calculated using generalized linear models in R adjusting for batch and position effects.

### Data analyses

For the analyses of fear conditioning, the independent variables in the analyses were *COMT* Genotype (three levels: Met/Met, Val/Met, Val/Val) and PTSD Diagnosis (two levels: PTSD−, PTSD+). The dependent variables were fear-potentiated startle and US-expectancy ratings. Fear-potentiated startle was calculated by subtracting the startle response magnitude during each CS presentation from the startle magnitude to the NA trials, in order to control for individual differences in startle magnitude. Repeated measures analyses of covariance (RM ANCOVA) were used to test the effect of Block (three levels) and Trial Type (two levels: CS+, CS−) during acquisition as within-subjects variables and Genotype and PTSD Diagnosis as between-subjects variables while co-varying for age, sex, race, and trauma history. Interaction effects of Block and Trial Type were followed up with RM ANCOVAs for comparing Trial Type within the last two Blocks of acquisition, when the fear conditioned effects are largest, as in our previous work (Norrholm et al., 2011). Further interactions with Trial Type were followed up by univariate analyses of covariance (ANCOVA) separately for each CS. Contingency awareness was analyzed by comparing US-expectancy ratings of each Trial Type with a Repeated Measures ANCOVA with Genotype and PTSD as between-groups factors. Extinction was analyzed the same way using RM ANCOVA, but with Block [three levels: early, mid, late, diving the six blocks into three bins consistent with our previous work (Norrholm et al., 2011; Glover et al., 2012)] as a within-subjects variable and Genotype and PTSD Diagnosis as between-group variables, using the above covariates. For the analyses of methylation, we first examined which CpG sites were most associated with genotype using a regression analysis in R correcting for multiple comparisons with a Bonferroni adjustment. Due to our previous findings of impaired fear inhibition to the CS−, we also completed a regression analysis of CpG sites were associated with fear-potentiated startle to the CS−. We then also examined the association between methylation of genotype-associated CpG sites and fear-potentiated startle to the CS−. Methylation was then also analyzed in an ANCOVA with genotype and PTSD a between-groups factors. Finally, in order to examine the association of methylation level and fear inhibition, we categorized individuals into High or Low methylation groups using a median split of the methylation beta values, and examined the effect of methylation level and PTSD diagnosis on fear-potentiated startle to the CS−. We also performed a step wise regression analysis with demographics, trauma history, PTSD, and continuous levels of methylation, to examine the independent contributions of each variable to fear inhibition.

## Results

### Participant characteristics

The study included 270 unrelated participants with varying degrees of trauma exposure (Table 1), of which 98 met criteria for PTSD (PTSD+ group), and 172 did not meet criteria for PTSD (PTSD− group). Of the total sample, 30 participants had the Met/Met genotype (21 without PTSD, 9 with PTSD), 94 participants had the Met/Val genotype (62 without PTSD, and 32 with PTSD), 145 had the Val/Val genotype (89 without PTSD, 57 with PTSD), χ^2^ = 0.55, ns. The demographic information is presented in Table 1. As expected, the PTSD+ and PTSD− groups were different in level of trauma exposure and PTSD symptoms; however, the genotype groups were matched on PTSD symptoms, although the Val/Met group reported slightly higher levels of trauma. Demographic data and trauma levels were entered as covariates in univariate analyses of variance. Table 2 shows the percent of different types of traumas experienced by individuals within each genotype group. The Val/Met genotype group had a greater likelihood of experiencing a serious injury, being attacked without a weapon, and having forced sexual contact after age 17.

**Table 1:** **Demographic characteristics, trauma level, and PTSD symptom severity across PTSD and *COMT* genotype groups**.

	PTSD diagnosis		COMT rs4680 genotype		
	PTSD+ (*n* = 98)	PTSD− (*n* = 172)	Met/Met (*n* = 30)	Val/Met (*n* = 94)	Val/Val (*n* = 146)
**DEMOGRAPHICS**					
Sex (% female)	67.3	60.7	73.3	54.3	66.4
Race (% AA)	90.8	87.9	80.0	79.8	96.6
Age (M, SD)	40.3 (11.4)	37.6 (12.9)	38.2 (12.1)	38.3 (12.2)	38.8 (12.7)
**TRAUMA EXPOSURE**					
TEI (M, SD)	4.6 (2.5)**	2.4 (2.2)	2.7 (2.3)	3.8 (2.9)*	3.0 (2.2)
**PTSD SYMPTOMS**					
PSS total (M, SD)	27.4 (9.5)**	7.1 (7.0)	12.8 (12.4)	13.6 (12.2)	14.0 (13.6)
PSS re-experiencing	7.0 (3.8)**	1.7 (2.5)	3.0 (3.7)	3.3 (3.7)	3.5 (4.2)
PSS avoidance	11.3 (4.3)**	2.8 (3.4)	5.9 (6.0)	5.3 (5.3)	5.6 (5.8)
PSS hyper-arousal	9.2 (3.6)**	2.8 (3.1)	3.8 (4.2)	5 (4.8)	4.9 (4.7)

* p < 0.05; ** p < 0.01.

**Table 2:** **Percent exposure to different trauma types across the *COMT* genotypes**.

Type of trauma exposure	Met/Met *N* (%)	Val/Met *N* (%)	Val/Val *N* (%)	*X*^2^
Natural disaster	9 (34.6)	32 (38.6)	42 (31.6)	1.11, ns
Serious accident or injury	11 (45.8)	48 (59.3)	53 (40.2)	7.38, *p* = 0.025
Sudden life-threatening illness	4 (15.4)	14 (17.5)	29 (21.6)	0.88, ns
Military combat	0 (0.0)	3 (3.8)	1 (0.7)	3.25, ns
Close friend or family member murdered	5 (19.2)	16 (20.3)	19 (14.2)	1.45, ns
Attacked with weapon	9 (34.6)	34 (44.7)	39 (30.7)	4.09, ns
Attacked without weapon	8 (30.8)	34 (43.0)	30 (22.7)	9.64, *p* = 0.008
Violence between parents or caregivers	8 (30.8)	28 (35.4)	52 (39.1)	0.77, ns
Childhood physical abuse	6 (23.1)	28 (35.4)	38 (29.7)	2.42, ns
Sexual contact before age 13	7 (26.9)	20 (25.3)	37 (28.0)	0.19, ns
Forced sexual contact between 14 and 17	6 (23.1)	9 (11.4)	17 (12.9)	2.39, ns
Forced sexual contact after age 17	0 (0.0)	15 (19.0)*	17 (12.8)	6.17, *p* = 0.046

* p < 0.05.

### Fear-potentiated startle

**Figure 2:**
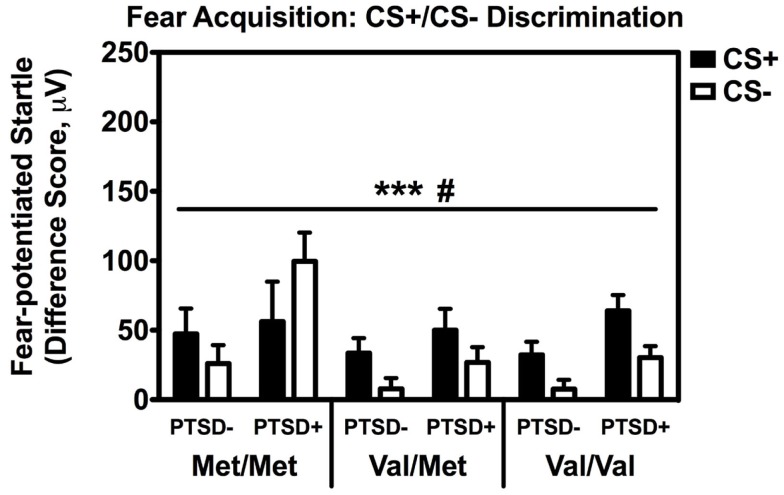
**Fear-potentiated startle during late fear acquisition across *COMT* genotype, PTSD diagnosis, and trial type**. There was a significant main effect of PTSD Diagnosis, *F*(2,258) = 6.80, *p* = 0.01, and an interaction effect of Genotype × Trial Type, *F*(2,258) = 3.13, *p* < 0.05. Covariates included in the analyses were age, sex, race, and trauma history. ***p < 0.001, ^#^*p* < 0.05.

Repeated measures analyses of covariance of fear-potentiated startle with Block and Trial Type as within-subject factors and Genotype and PTSD Diagnosis as between-groups variables, with age, sex, race, and trauma history as covariates, revealed a significant main effect of Block [*F*(2,514) = 18.24, *p* < 0.001], main effect of PTSD Diagnosis [*F*(1,257) = 6.45, *p* = 0.01], a significant two-way Block × Trial Type interaction [*F*(2,514) = 3.15, *p* < 0.05], and a significant two-way Genotype × Trial Type interaction, *F*(2,257) = 3.23, *p* < 0.05. We then performed another RM ANCOVA during late acquisition, with Trial Type as the within-subjects variable, and Genotype and PTSD Diagnosis as the between-groups variables. We found a significant main effect of PTSD Diagnosis, *F*(2,258) = 6.80, *p* = 0.01, and an interaction effect of Genotype × Trial Type, *F*(2,258) = 3.13, *p* < 0.05 (see Figure 2). A follow-up RM ANCOVA analysis of Trial Type within each Genotype revealed significantly higher fear-potentiated startle to the CS+ than the CS− in the Val/Met group, *F*(1,93) = 17.47, *p* = 0.00007, and the Val/Val group, *F*(1,145) = 21.58, *p* = 0.000008, but not in the Met/Met group, *F*(1,29) = 0.05, ns. As seen in Figure 2, the lack of discrimination between CS+ and CS− in the Met/Met group was largely due to the PTSD+ individuals. In order to ensure that this lack of discrimination was not due to impaired cognitive learning of the CS contingencies in the Met/Met group, we repeated the above analyses with US-expectancy ratings as the dependent variable. We conducted RM ANCOVA of US-expectancy across Block and CS+ vs. CS−, with Genotype and PTSD as between-groups factors. This analysis revealed a significant interaction of Block and Trial Type, *F*(2,310) = 4.08, *p* < 0.05. Looking at late acquisition, we found significantly higher US expectancies on the CS+ than the CS− across all groups after controlling for age, sex, race, and trauma history, *F*(1,157) = 24.60, *p* < 0.00001, but no main or interaction effects with Genotype or PTSD Diagnosis. This suggests that all groups were equally able to discriminate, at a conscious level, between the CS+ vs. CS− shapes.

**Figure 3:**
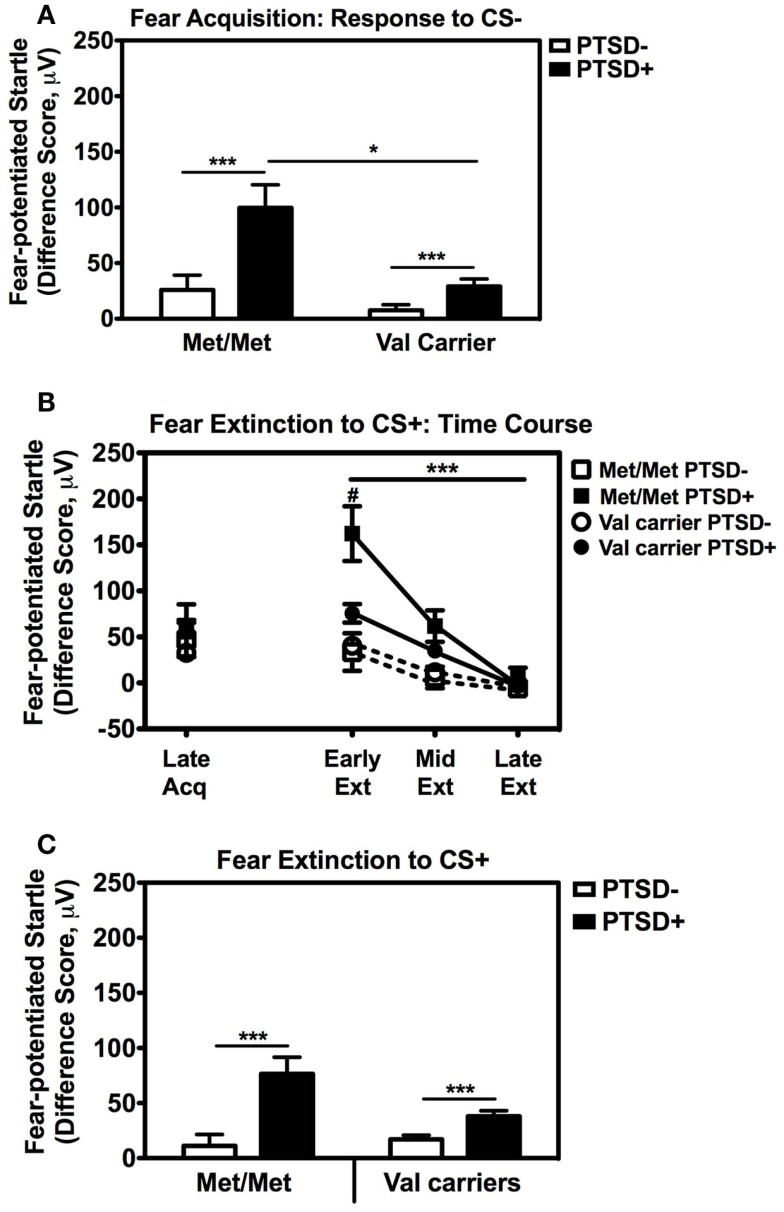
**Fear-potentiated startle during late acquisition and extinction across *COMT* genotype and PTSD diagnosis**. **(A)** Fear-potentiated startle to the CS− (safety signal) in the Met/Met PTSD+ group was higher than the Val carriers with PTSD, *F*(1,92) = 8.01, *p* = 0.006. Covariates included in the analyses were age, sex, race, and trauma history. Sample sizes: Met/Met PTSD− = 21; Val carriers PTSD− = 151; Met/Met PTSD+ = 9; Val carriers PTSD+ = 89. ***p < 0.001, *p < 0.01. **(B)** Fear-potentiated startle to the CS+ (danger signal) during late acquisition and early, mid, and late extinction across Genotype and PTSD Diagnosis. We found a significant linear effect of Extinction, *F*(2,474) = 29.87, *p* < 0.00001, and a significant interaction of Extinction and PTSD Diagnosis, *F*(2,474) = 10.36, *p* < 0.00001, an interaction of Extinction and Genotype, *F*(2,474) = 3.54, *p* < 0.05, as well as a three-way interaction of Extinction, Genotype, and PTSD Diagnosis, *F*(2,474) = 3.98, *p* = 0.03. Covariates included in the analyses were age, sex, race, and trauma history. Sample sizes: Met/Met PTSD− = 18; Val carriers PTSD− = 142; Met/Met PTSD+ = 9; Val carriers PTSD+ = 80. ***p < 0.001, ^#^*p* < 0.05. **(C)** Fear-potentiated startle to the CS+ during extinction in the PTSD+ group was higher than the control group (PTSD−) in both genotypes, *F*(1,238) = 19.18, *p* = 0.00001. Covariates included in the analyses were age, sex, race, and trauma history. Sample sizes: Met/Met PTSD− = 18; Val carriers PTSD− = 142; Met/Met PTSD+ = 9; Val carriers PTSD+ = 80. ***p < 0.001.

We also followed up the above Genotype × Trial Type interaction effect by analyzing the effects of Genotype and PTSD Diagnosis within each Trial Type separately, while controlling for potential covariates. A univariate ANCOVA of fear-potentiated startle to the CS+ (danger signal), with sex, age, race, and trauma levels entered as covariates did not show any main or interaction effects. However, the same analysis of fear-potentiated startle to the CS− revealed a significant main effect of Genotype, *F*(2,258) = 5.94, *p* = 0.003, a main effect of PTSD Diagnosis, *F*(1,258) = 13.39, *p* = 0.0003, but no interaction effect between Genotype and PTSD, *F*(2,258) = 2.10, *p* > 0.1. In all genotype groups, fear to the CS− was higher in the PTSD+ group compared to the PTSD− group (see Figure 3A). A Tukey HSD *post hoc* analysis of the main effect of Genotype, showed that fear-potentiated startle to the CS− was higher in the Met/Met group than either the Val/Met group (*p* < 0.02), or the Val/Val group (*p* < 0.05). As there were no significant differences between the two Val genotypes, the Val/Met and Val/Val groups were collapsed in the further analyses described below. When we examined fear-potentiated startle to the safety signal within each diagnostic group with the Val/Met and Val/Val genotypes collapsed, we found that it was highest in the PTSD+ Met/Met genotype carriers compared to Val carriers, *F*(1,92) = 8.01, *p* = 0.006 (see Figure 3A). In order to further control for the effects of race, we stratified the sample by race and repeated the significant analyses in only African American individuals (*n* = 240); the main effect of Genotype was still significant, *F*(2,229) = 6.22, *p* = 0.002. Furthermore, fear-potentiated startle was again highest in the PTSD+ Met/Met group compared to Val allele carriers, *F*(1,84) = 7.91, *p* = 0.006.

These results indicate that Met158 homozygotes have higher physiological fear responses to safety signals, and that this elevation may be further enhanced in those with PTSD. In our previous studies, fear-potentiated startle to the CS− during acquisition predicted the degree of CS+ extinction (Norrholm et al., 2011). Therefore, we examined extinction to the CS+ with a RM ANCOVA of Extinction (three levels: Early, Mid, Late) with Genotype (two levels: Met/Met, Val carriers) and PTSD Diagnosis (two levels; PTSD+, PTSD−) as between-subjects factors, with the same covariates as above. We found a significant linear effect of Extinction, *F*(2,474) = 29.87, *p* < 0.00001, and a significant interaction of Extinction and PTSD Diagnosis, *F*(2,474) = 10.36, *p* < 0.00001, an interaction of Extinction and Genotype, *F*(2,474) = 3.54, *p* < 0.05, as well as a three-way interaction of Extinction, Genotype, and PTSD Diagnosis, *F*(2,474) = 3.98, *p* = 0.03. Figure 3B shows the extinction curves for the Genotype and PTSD groups over time. The RM ANCOVA also indicated a significant main effect of PTSD Diagnosis on extinction, *F*(1,238) = 19.18, *p* = 0.00001 (see Figure 3C), and an interaction effect of Genotype and PTSD, *F*(1,238) = 5.49, *p* = 0.02, with the PTSD+ Met/Met genotype group showing the highest levels of fear during extinction (Figure 3C). These results indicate that fear inhibition to the safety signal during acquisition and extinction are impaired in Met/Met carriers with PTSD. As in our previous work, we found a high correlation between fear-potentiated startle to the CS− and extinction to the CS+ in this data set (*r* = 0.56, *p* < 0.00001). Thus, we next examined *COMT* methylation with respect to CS− only.

### Methylation

**Figure 4:**
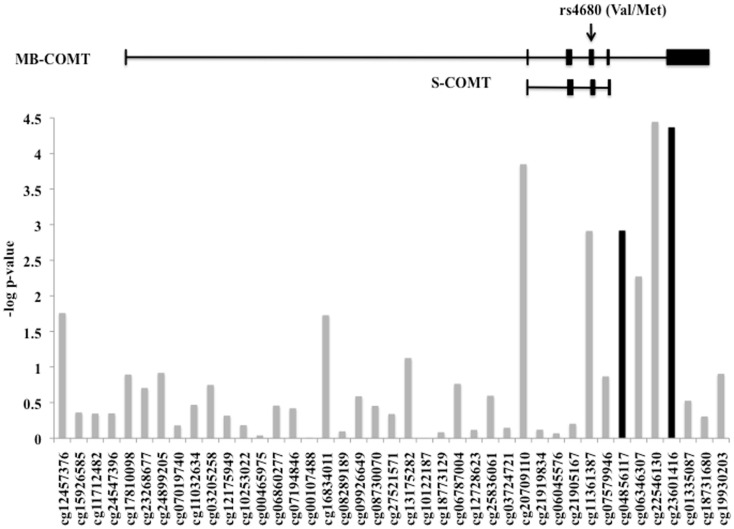
**DNA methylation of the *COMT* gene**. Plot of the CpG probes (*x*-axis) vs. the negative log for the *p*-values for association with genotype-dependent methylation. The genomic structure of across *COMT* is indicated along with the location of the rs4680 SNP. Four CpG sites associated with *COMT* genotype, and two of these (in black) were associated with fear-potentiated startle to the CS−. Sample size: total *N* = 185.

The level of DNA methylation of 41 CpG sites that spanned the *COMT* gene was available for 185 participants. The *COMT* genotype was associated with DNA methylation of four CpG sites in the promoter region of *COMT* after correction for multiple comparisons and adjusting for batch effects, age, sex, and race (*p* < 0.0012, Figure 4). Of these, two CpG sites were also independently associated with fear-potentiated startle to the CS− (*p* < 0.029; see Figure 4, black bars). In each of these cases, the Met/Met genotype was associated with increased methylation. A multivariate ANCOVA of methylation levels of these two CpG sites with genotype and PTSD as between-groups variables, and age, sex, race, and trauma as covariates, revealed a significant omnibus effect of genotype, Wilks’ Lambda *F*(8,348) = 6.78, *p* = 0.00004, but no effect of PTSD or interaction with PTSD. The main effect of genotype was significant at each site (cg23061416, *F*(2,174) = 13.25, *p* = 0.000004 and cg04856117, *F*(2,174) = 10.30, *p* = 0.00006). Tukey HSD *post hoc* tests showed an effect of Met allele number on methylation at each site (i.e., Met/Met > Val/Met > Val/Val).

**Figure 5:**
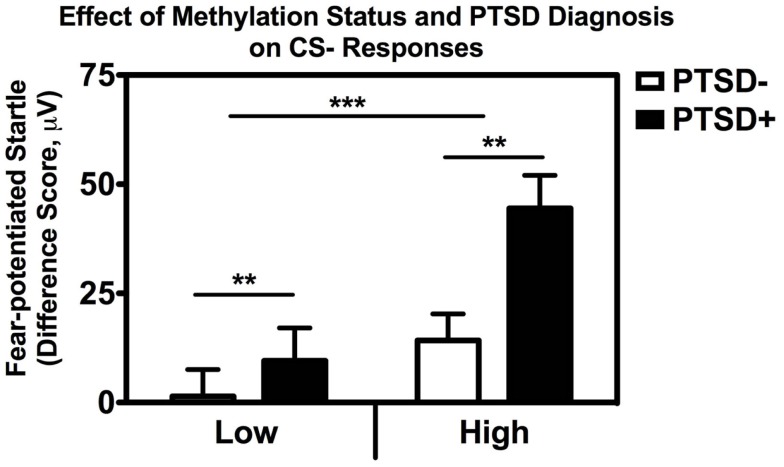
**Association between *COMT* methylation, genotype, and startle**. Fear-potentiated startle to the CS− (safety signal) across high vs. low methylation groups and PTSD diagnosis. There was a significant main effect of methylation level, *F*(1,176) = 11.73, *p* = 0.001, and PTSD *F*(1,176) = 9.66, *p* = 0.002, after co-varying for sex, age, race, and trauma history. Sample sizes: low methylation PTSD− = 54; high methylation PTSD− = 56; low methylation PTSD+ = 37; high methylation PTSD+ = 37. **p < 0.01; ***p < 0.001.

In order to examine the interaction between methylation and PTSD with respect to fear inhibition, we used a median split to divide the subjects into those with High and Low methylation levels at this same CpG site, and performed a univariate ANCOVA with fear-potentiated startle to the CS− as the dependent variable and age, sex, race, and trauma as covariates. We found significant main effects of PTSD, *F*(1,176) = 9.66, *p* = 0.002, and methylation, *F*(1,176) = 11.73, *p* = 0.001, in that participants with High methylation levels had greater levels of fear to the safety signal compared to participants with Low methylation levels (Figure 5). We again repeated the analysis in an exclusively African American sample, and obtained the same results: those with High methylation had higher levels of fear-potentiated startle to the CS− compared to those with Low methylation levels, *F*(1,176) = 10.56, *p* = 0.001.

Finally, in order to parse out the unique contribution of methylation to impaired fear inhibition, we conducted a stepwise regression analysis with fear-potentiated startle to the safety signal as the outcome variable, entering demographics in the first step, PTSD in the second step, and methylation at CpG site cg23061416 in the third step. The overall model was significant, *F*(5,180) = 5.354.57, *p* < 0.0001, with methylation alone accounting for 4.2% of the variance in startle to the safety signal after controlling for PTSD, *F*_change_(1,180) = 8.68, *p* = 0.004. These analyses suggest that higher methylation levels are associated with higher fear to the safety signal, and impaired fear inhibition.

## Discussion

The current fear-potentiated startle study, based on previous studies of COMT genotype and fear, anxiety, and trauma, initially explored the relationship between the COMT Val^158^Met polymorphism and conditioned fear responses in an inner-city population of traumatized individuals. We hypothesized that *COMT* Met/Met genotype, associated with less efficient COMT function in prefrontal cortex, would be related to impaired fear inhibition in PTSD subjects. The main findings of this study are as follows: (1) traumatized individuals with PTSD and the Met/Met genotype displayed higher fear to the CS− (safety signal) as compared to carriers of the Val allele, (2) PTSD was associated with impaired fear extinction, which was most impaired in Met/Met subjects with PTSD, (3) the Met/Met genotype-associated with DNA methylation at four CpG sites; methylation at two of these sites was also associated with fear-potentiated startle to the CS−, and (4) stepwise regression analysis with fear-potentiated startle to the CS− as the outcome variable revealed that methylation accounted for a significant degree of the variance in startle to the CS− after controlling for trauma exposure. The results of this study suggest that, in addition to *COMT* Val^158^Met genotype, higher methylation at these CpG sites in the promoter region of *COMT* is associated with impaired fear inhibition in an established fear conditioning paradigm (Jovanovic et al., 2010b; Norrholm et al., 2011).

Fear inhibition may be a robust intermediate phenotype in PTSD. These data suggest that altered COMT function, at the genetic, epigenetic, and gene expression levels, may result in disrupted catecholamine metabolism and thus disrupted regulation of fear inhibition. Our current findings are consistent with previous reports suggesting an association between the Met/Met genotype and greater limbic responsiveness (Smolka et al., 2005; Drabant et al., 2006; Heinz and Smolka, 2006), susceptibility to anxiety disorders (Enoch et al., 2003; Woo et al., 2004; Olsson et al., 2005), response to trauma exposure, and the development of PTSD (Kolassa et al., 2010; Valente et al., 2011).

We found that the Met/Met genotype was associated with methylation at four CpG sites in the promoter region of *COMT* and that two of these sites were associated with fear-potentiated startle to the CS− (safety signal). In recent years, it has become increasingly evident that the etiology of many psychiatric disorders, including PTSD, involves the complex interaction between genetic and environmental factors and that the latter can influence epigenetic mechanisms including DNA methylation (Tsankova et al., 2007). As noted by Jones and Takai (2001), methylation of CpG sites in the promoter region of a gene represents a primary form of epigenetic modification and an important mechanism for influencing gene expression (Jones and Takai, 2001). It has been suggested that DNA methylation serves as a critical substrate upon which gene × environment interactions contribute to one’s response to environmental stressors and, ultimately, risk for the development of psychiatric illness (Abdolmaleky et al., 2004). This is especially important to consider with regard to the current study.

Posttraumatic stress disorder is a psychiatric illness that is precipitated by exposure to a traumatic event (e.g., an environmental stressor) and the development and severity of symptoms appears to be strongly influenced by gene × environment interactions (Kilpatrick et al., 2007; Koenen, 2007; Norrholm and Ressler, 2009; Skelton et al., 2012). The present study demonstrates that the presence of the Met/Met genotype (a putative PTSD risk factor) coupled with CpG site methylation (an epigenetic mechanism) is associated with impaired fear inhibition; a phenomenon that has been linked to the fear-related symptoms of PTSD (Jovanovic et al., 2009, 2010a). The association of the Met/Met genotype and risk for PTSD has been found in studies with larger sample sizes (e.g., Kolassa et al., 2010), however, in the current study the genotype did not appear to confer risk for the disorder *per se* given that approximately a third of each genotype met criteria for PTSD, rather this genotype was linked to a physiological phenotype previously associated with PTSD. One of the benefits of using intermediate, neurobiological phenotypes such as fear inhibition is that the genotypic risk can be assessed in a smaller sample size. It is important to note that the impaired fear inhibition phenotype from our earlier studies was replicated with both higher fear to the safety signal and impaired fear extinction in a much larger sample of PTSD patients in the current study. Therefore, the exaggerated “fear load” in PTSD appears to be a robust physiological phenotype (Norrholm et al., 2011; Valente et al., 2011; Fani et al., 2012 #24384). As shown in Figure 3B, higher fear load in the Met/Met PTSD+ group was associated with slower extinction, a finding that is consistent with our previous research on extinction in PTSD (Norrholm et al., 2011; Glover et al., 2012).

The *COMT* Val^158^Met polymorphism has been widely examined with regard to susceptibility and symptom expression of psychiatric illnesses (Witte and Flöel, 2012). More recently, methylation of CpG sites in the promoter region of *COMT* has been linked to psychiatric diagnoses such as schizophrenia (Murphy et al., 2005; Abdolmaleky et al., 2006), bipolar disorder (Abdolmaleky et al., 2006), and nicotine dependence (Xu et al., 2010) as well as prefrontal cognition and activity (Ursini et al., 2011). This is the first report of an association between methylation of the *COMT* promoter, fear responses, and PTSD symptoms. Our data suggests that differential CpG methylation levels may be independently associated with PTSD symptoms. Separately, at the post-translational level, the Val^158^Met polymorphism is most associated with the decreased COMT catabolic enzyme function. Together these data suggest that for proteins that are particularly critical to cognitive processing, understanding their differential regulation at multiple biological levels may prove fruitful in determining risk for psychiatric illness.

The *COMT* Val158Met polymorphism has attracted significant interest in neuroscience and psychiatry, primarily because of the enzyme’s key role in metabolizing dopamine and its robust association with function of the prefrontal cortex (Mier et al., 2010). While much of the research regarding the contribution of this polymorphism to the etiology of psychiatric illness has focused on dopaminergic systems, one cannot discount the potential role of COMT-mediated alterations in noradrenergic and adrenergic signaling. For example, similar to its effect on dopamine signaling, the low activity, homozygous Met genotype has been associated with higher plasma concentrations (Jung et al., 2012) and higher synaptic levels of norepinephrine (Tunbridge, 2010).

Similar to other studies of DNA methylation status, an inherent limitation of the current study is the extraction of DNA from peripheral blood as opposed to brain tissue and, as such, one cannot completely rule out the possibility that divergent methylation processes occur in peripheral lymphocytes vs. brain tissue. However, as discussed by Xu et al. (2010), numerous genetic studies have employed peripheral blood samples and empirical evidence suggests that there is a high correlation between expression patterns observed in blood cells as compared to brain cells (Gladkevich et al., 2004; Davies et al., 2012).

An advantage of the current study is the access to a large traumatized civilian population; a population size that exceeds many of the extant studies in this area. However, due to the rarity of the Met/Met genotype, our relatively large sample size (270 participants) yielded 30 Met homozygotes with 9 also having a diagnosis of PTSD. This frequency of Met allele carriers is consistent with other studies of the *COMT* Val^158^Met polymorphism and anxiety disorders (Montag et al., 2008; Lonsdorf et al., 2009; Kolassa et al., 2010; Valente et al., 2011).

In summary, we have shown in a highly traumatized sample at significant environmental risk for PTSD, that differential risk vs. resilience is, in part, mediated by multiple levels of molecular regulation on COMT function. Although our data do not directly address the downstream mechanism, multiple lines of evidence suggest that COMT regulates synaptic catecholamine levels, particularly dopamine, and that this can closely modulate cortical functioning. Our data suggest that at the level of protein function, as well as with differential epigenetic and transcriptional control, *COMT* regulation may be an important mediator of fear processing, particularly fear inhibition. Thus multiple mechanisms of regulating neurotransmitter catabolism may interact in differentiating cognitive processes underlying PTSD following trauma exposure.

## Conflict of Interest Statement

The authors declare that the research was conducted in the absence of any commercial or financial relationships that could be construed as a potential conflict of interest.

## References

[B1] Abdolmaleky H. M. Cheng K. H. Faraone S. V. Wilcox M. Glatt S. J. Gao F. (2006). Hypomethylation of MB-COMT promoter is a major risk factor for schizophrenia and bipolar disorder. Hum. Mol. Genet. 15, 3132–3145 10.1093/hmg/ddl253 16984965 PMC2799943

[B2] Abdolmaleky H. M. Smith C. L. Faraone S. V. Shafa R. Stone W. Glatt S. J. (2004). Methylomics in psychiatry: modulation of gene-environment interactions may be through DNA methylation. Am. J. Med. Genet. B Neuropsychiatr. Genet. 127B, 51–59 10.1002/ajmg.b.20142 15108180

[B3] Amstadter A. B. Nugent N. R. Koenen K. C. (2009). Genetics of PTSD: fear conditioning as a model for future research. Psychiatr. Ann. 39, 358–367 10.3928/00485713-20090526-01 19779593 PMC2749314

[B4] Armbruster D. Mueller A. Strobel A. Lesch K. P. Kirschbaum C. Brocke B. (2011). Variation in genes involved in dopamine clearance influence the startle response in older adults. J. Neural Transm. 118, 1281–1292 10.1007/s00702-011-0625-6 21445667

[B5] Armbruster D. Mueller A. Strobel A. Lesch K. P. Brocke B. Kirschbaum C. (2012). Children under stress – COMT genotype and stressful life events predict cortisol increase in an acute social stress paradigm. Int. J. Neuropsychopharmacol. 15, 1229–1239 10.1017/S1461145711001763 22152146

[B6] Bilder R. M. Volavka J. Lachman H. M. Grace A. A. (2004). The catechol-O-methyltransferase polymorphism: relations to the tonic-phasic dopamine hypothesis and neuropsychiatric phenotypes. Neuropsychopharmacology 29, 1943–1961 10.1038/sj.npp.1300542 15305167

[B7] Binder E. B. Bradley R. G. Liu W. Epstein M. Deveau T. Mercer K. B. (2008). Association of FKBP5 polymorphisms and child abuse with risk of posttraumatic stress disorder symptoms in adults. JAMA 299, 1291–1305 10.1001/jama.299.11.1291 18349090 PMC2441757

[B8] Chen J. Lipska B. K. Halim N. Ma Q. D. Matsumoto M. Melhem S. (2004). Functional analysis of genetic variation in catechol-O-methyltransferase (COMT): effects on mRNA, protein, and enzyme activity in postmortem human brain. Am. J. Hum. Genet. 75, 807–821 10.1086/425588 15457404 PMC1182110

[B9] Davies M. N. Volta M. Pidsley R. Lunnon K. Dixit A. Lovestone S. (2012). Functional annotation of the human brain methylome identifies tissue-specific epigenetic variation across brain and blood. Genome Biol. 13, R43 10.1186/gb-2012-13-6-r43 22703893 PMC3446315

[B10] Drabant E. M. Hariri A. R. Meyer-Lindenberg A. Munoz K. E. Mattay V. S. Kolachana B. S. (2006). Catechol O-methyltransferase val158met genotype and neural mechanisms related to affective arousal and regulation. Arch. Gen. Psychiatry 63, 1396–1406 10.1001/archpsyc.63.12.1396 17146014

[B11] Enoch M. A. Xu K. Ferro E. Harris C. R. Goldman D. (2003). Genetic origins of anxiety in women: a role for a functional catechol-O-methyltransferase polymorphism. Psychiatr. Genet. 13, 33–41 10.1097/00041444-200303000-00006 12605099

[B12] Fani N. Tone E. B. Phifer J. Norrholm S. D. Bradley B. Ressler K. J. (2012). Attention bias toward threat is associated with exaggerated fear expression and impaired extinction in PTSD. Psychol. Med. 42, 533–543 10.1017/S0033291711001565 21854700 PMC3690118

[B13] Gladkevich A. Kauffman H. F. Korf J. (2004). Lymphocytes as a neural probe: potential for studying psychiatric disorders. Prog. Neuropsychopharmacol. Biol. Psychiatry 28, 559–576 10.1016/j.pnpbp.2004.01.009 15093964

[B14] Glover E. M. Jovanovic T. Mercer K. B. Kerley K. Bradley B. Ressler K. J. (2012). Estrogen levels are associated with extinction deficits in women with posttraumatic stress disorder. Biol. Psychiatry 72, 19–24 10.1016/j.biopsych.2012.04.007 22502987 PMC3675159

[B15] Gogos J. A. Morgan M. Luine V. Santha M. Ogawa S. Pfaff D. (1998). Catechol-O-methyltransferase-deficient mice exhibit sexually dimorphic changes in catecholamine levels and behavior. Proc. Natl. Acad. Sci. U.S.A. 95, 9991–9996 10.1073/pnas.95.17.9991 9707588 PMC21449

[B16] Heinz A. Smolka M. N. (2006). The effects of catechol O-methyltransferase genotype on brain activation elicited by affective stimuli and cognitive tasks. Rev. Neurosci. 17, 359–367 16878403 10.1515/revneuro.2006.17.3.359

[B17] Jones P. A. Takai D. (2001). The role of DNA methylation in mammalian epigentics. Science 293, 1068–1070 10.1126/science.1063852 11498573

[B18] Jovanovic T. Norrholm S. D. (2011). Neural mechanisms of impaired fear inhibition in posttraumatic stress disorder. Front. Behav. Neurosci. 5:44 10.3389/fnbeh.2011.00044 21845177 PMC3145245

[B19] Jovanovic T. Norrholm S. D. Blanding N. Q. Davis M. Duncan E. Bradley B. (2010a). Impaired fear inhibition is a biomarker of PTSD but not depression. Depress. Anxiety 27, 244–251 10.1002/da.20663 20143428 PMC2841213

[B20] Jovanovic T. Norrholm S. D. Blanding N. Q. Phifer J. E. Weiss T. Davis M. (2010b). Fear potentiation is associated with hypothalamic-pituitary-adrenal axis function in PTSD. Psychoneuroendocrinology 35, 846–857 10.1016/j.psyneuen.2009.11.009 20036466 PMC2875386

[B21] Jovanovic T. Norrholm S. D. Fennell J. E. Keyes M. Fiallos A. Myers K. M. (2009). Posttraumatic stress disorder may be associated with impaired fear inhibition: relation to symptom severity. Psychiatry Res. 167, 151–160 10.1016/j.psychres.2007.12.014 19345420 PMC2713500

[B22] Jovanovic T. Norrholm S. D. Keyes M. Fiallos A. Jovanovic S. Myers K. M. (2006). Contingency awareness and fear inhibition in a human fear-potentiated startle paradigm. Behav. Neurosci. 120, 995–1004 10.1037/0735-7044.120.5.995 17014251 PMC3740393

[B23] Jovanovic T. Ressler K. J. (2010). How the neurocircuitry and genetics of fear inhibition may inform our understanding of PTSD. Am. J. Psychiatry 167, 648–662 10.1176/appi.ajp.2009.09071074 20231322 PMC3603297

[B24] Jung Y. H. Kang D. H. Byun M. S. Shim G. Kwon S. J. Jang G. E. (2012). Influence of brain-derived neurotrophic factor and catechol O-methyl transferase polymorphisms on effects of meditation on plasma catecholamines and stress. Stress 15, 97–104 21790467 10.3109/10253890.2011.592880

[B25] Kilpatrick D. G. Koenen K. C. Ruggiero K. J. Acierno R. Galea S. Resnick H. S. (2007). The serotonin transporter genotype and social support and moderation of posttraumatic stress disorder and depression in hurricane-exposed adults. Am. J. Psychiatry 164, 1693–1699 10.1176/appi.ajp.2007.06122007 17974934

[B26] Koenen K. C. (2007). Genetics of posttraumatic stress disorder: review and recommendations for future studies. J. Trauma Stress 20, 737–750 10.1002/jts.20191 17955543

[B27] Kolassa I. T. Kolassa S. Ertl V. Papassotiropoulos A. De Quervain D. J. (2010). The risk of posttraumatic stress disorder after trauma depends on traumatic load and the catechol-o-methyltransferase Val(158)Met polymorphism. Biol. Psychiatry 67, 304–308 10.1016/j.biopsych.2009.10.009 19944409

[B28] Lewis D. A. Melchitzky D. S. Sesack S. R. Whitehead R. E. Auh S. Sampson A. (2001). Dopamine transporter immunoreactivity in monkey cerebral cortex: regional, laminar, and ultrastructural localization. J. Comp. Neurol. 432, 119–136 10.1002/cne.1092 11241381

[B29] Lonsdorf T. B. Weike A. I. Nikamo P. Schalling M. Hamm A. O. Ohman A. (2009). Genetic gating of human fear learning and extinction: possible implications for gene-environment interaction in anxiety disorder. Psychol. Sci. 20, 198–206 10.1111/j.1467-9280.2009.02280.x 19175757

[B30] Matsumoto M. Weickert C. S. Akil M. Lipska B. K. Hyde T. M. Herman M. M. (2003). Catechol O-methyltransferase mRNA expression in human and rat brain: evidence for a role in cortical neuronal function. Neuroscience 116, 127–137 10.1016/S0306-4522(02)00556-0 12535946

[B31] Institute of Medicine (2008). Treatment of Posttraumatic Stress Disorder: An Assessment of the Evidence. Washington, DC: The National Academies Press

[B32] Mier D. Kirsch P. Meyer-Lindenberg A. (2010). Neural substrates of pleiotropic action of genetic variation in COMT: a meta-analysis. Mol. Psychiatry 15, 918–927 10.1038/mp.2009.36 19417742

[B33] Milad M. R. Orr S. P. Lasko N. B. Chang Y. Rauch S. L. Pitman R. K. (2008). Presence and acquired origin of reduced recall for fear extinction in PTSD: results of a twin study. J. Psychiatr. Res. 42, 515–520 10.1016/j.jpsychires.2008.01.017 18313695 PMC2377011

[B34] Montag C. Buckholtz J. W. Hartmann P. Merz M. Burk C. Hennig J. (2008). COMT genetic variation affects fear processing: psychophysiological evidence. Behav. Neurosci. 122, 901–909 10.1037/0735-7044.122.4.901 18729643

[B35] Murphy B. C. O’Reilly R. L. Singh S. M. (2005). Site-specific cytosine methylation in S-COMT promoter in 31 brain regions with implications for studies involving schizophrenia. Am. J. Med. Genet. B Neuropsychiatr. Genet. 133B, 37–42 10.1002/ajmg.b.30134 15635661

[B36] Norrholm S. D. Jovanovic T. Olin I. W. Sands L. A. Karapanou I. Bradley B. (2011). Fear extinction in traumatized civilians with posttraumatic stress disorder: relation to symptom severity. Biol. Psychiatry 69, 556–563 10.1016/j.biopsych.2010.09.013 21035787 PMC3052965

[B37] Norrholm S. D. Jovanovic T. Vervliet B. Myers K. M. Davis M. Rothbaum B. O. (2006). Conditioned fear extinction and reinstatement in a human fear-potentiated startle paradigm. Learn. Mem. 13, 681–685 10.1101/lm.393906 17142300 PMC3746591

[B38] Norrholm S. D. Ressler K. J. (2009). Genetics of anxiety and trauma-related disorders. Neuroscience 164, 272–287 10.1016/j.neuroscience.2009.06.036 19540311 PMC2760665

[B39] Norrholm S. D. Vervliet B. Jovanovic T. Boshoven W. Myers K. M. Davis M. (2008). Timing of extinction relative to acquisition: a parametric analysis of fear extinction in humans. Behav. Neurosci. 122, 1016–1030 10.1037/a0012604 18823159 PMC3731450

[B40] Olsson C. A. Anney R. J. Lotfi-Miri M. Byrnes G. B. Williamson R. Patton G. C. (2005). Association between the COMT Val158Met polymorphism and propensity to anxiety in an Australian population-based longitudinal study of adolescent health. Psychiatr. Genet. 15, 109–115 10.1097/00041444-200506000-00007 15900225

[B41] Sesack S. R. Hawrylak V. A. Matus C. Guido M. A. Levey A. I. (1998). Dopamine axon varicosities in the prelimbic division of the rat prefrontal cortex exhibit sparse immunoreactivity for the dopamine transporter. J. Neurosci. 18, 2697–2708 9502827 10.1523/JNEUROSCI.18-07-02697.1998 PMC6793120

[B42] Skelton K. Ressler K. J. Norrholm S. D. Jovanovic T. Bradley-Davino B. (2012). PTSD and gene variants: new pathways and new thinking. Neuropharmacology 62, 628–637 10.1016/j.neuropharm.2011.02.013 21356219 PMC3136568

[B43] Slifstein M. Kolachana B. Simpson E. H. Tabares P. Cheng B. Duvall M. (2008). COMT genotype predicts cortical-limbic D1 receptor availability measured with [11C]NNC112 and PET. Mol. Psychiatry 13, 821–827 10.1038/mp.2008.19 18317466

[B44] Smolka M. N. Schumann G. Wrase J. Grusser S. M. Flor H. Mann K. (2005). Catechol-O-methyltransferase val158met genotype affects processing of emotional stimuli in the amygdala and prefrontal cortex. J. Neurosci. 25, 836–842 10.1523/JNEUROSCI.1792-04.2005 15673663 PMC6725630

[B45] Tsankova N. Renthal W. Kumar A. Nestler E. J. (2007). Epigenetic regulation in psychiatric disorders. Nat. Rev. Neurosci. 8, 355–367 10.1038/nrm2162 17453016

[B46] Tunbridge E. Burnet P. W. Sodhi M. S. Harrison P. J. (2004a). Catechol-o-methyltransferase (COMT) and proline dehydrogenase (PRODH) mRNAs in the dorsolateral prefrontal cortex in schizophrenia, bipolar disorder, and major depression. Synapse 51, 112–118 10.1002/syn.10286 14618678

[B47] Tunbridge E. M. Bannerman D. M. Sharp T. Harrison P. J. (2004b). Catechol-o-methyltransferase inhibition improves set-shifting performance and elevates stimulated dopamine release in the rat prefrontal cortex. J. Neurosci. 24, 5331–5335 10.1523/JNEUROSCI.1124-04.2004 15190105 PMC6729311

[B48] Tunbridge E. M. (2010). The catechol-O-methyltransferase gene: its regulation and polymorphisms. Int. Rev. Neurobiol. 95, 7–27 10.1016/B978-0-12-381326-8.00002-8 21095457

[B49] Ursini G. Bollati V. Fazio L. Porcelli A. Iacovelli L. Catalani A. (2011). Stress-related methylation of the catechol-O-methyltransferase Val158 allele predicts human prefrontal cognition and activity. J. Neurosci. 31, 6692–6698 10.1523/JNEUROSCI.6631-10.2011 21543598 PMC6632869

[B50] Valente N. L. Vallada H. Cordeiro Q. Bressan R. A. Andreoli S. B. Mari J. J. (2011). Catechol-O-methyltransferase (COMT) val158met polymorphism as a risk factor for PTSD after urban violence. J. Mol. Neurosci. 43, 516–523 10.1007/s12031-010-9474-2 21080103

[B51] Witte A. V. Flöel A. (2012). Effects of COMT polymorphisms on brain function and behavior in health and disease. Brain Res. Bull. 88, 418–428 10.1016/j.brainresbull.2011.11.012 22138198

[B52] Woo J. M. Yoon K. S. Choi Y. H. Oh K. S. Lee Y. S. Yu B. H. (2004). The association between panic disorder and the L/L genotype of catechol-O-methyltransferase. J. Psychiatr. Res. 38, 365–370 10.1016/j.jpsychires.2004.01.001 15203287

[B53] Xu Q. Ma J. Z. Payne T. J. Li M. D. (2010). Determination of methylated CpG sites in the promoter region of catechol-O-methyltransferase (COMT) and their involvement in the etiology of tobacco smoking. Front. Psychiatry 1:16 10.3389/fpsyt.2010.00016 21423427 PMC3059640

